# Complement Nomenclature—Deconvoluted

**DOI:** 10.3389/fimmu.2019.01308

**Published:** 2019-06-07

**Authors:** Suzanne S. Bohlson, Peter Garred, Claudia Kemper, Andrea J. Tenner

**Affiliations:** ^1^Department of Microbiology and Immunology, Des Moines University, Des Moines, IA, United States; ^2^Laboratory of Molecular Medicine, Department of Clinical Immunology, Rigshospitalet University Hospital of Copenhagen, Copenhagen, Denmark; ^3^Laboratory of Molecular Immunology and the Immunology Center, National Heart, Lung, and Blood Institute (NHLBI), National Institutes of Health (NIH), Bethesda, MD, United States; ^4^Department of Molecular Biology and Biochemistry, Department of Neurobiology and Behavior, Department of Pathology and Laboratory Medicine, University of California, Irvine, Irvine, CA, United States

**Keywords:** complement, nomenclature, C1, C1q, C2, lectin pathway, collectins, clusterin

## Abstract

In 2014, specific recommendations for complement nomenclature were presented by the complement field. There remained some unresolved designations and new areas of ambiguity, and here we propose solutions to resolve these remaining issues. To enable rapid understanding of the intricate complement system and facilitate therapeutic development and application, a uniform nomenclature for cleavage fragments, pattern recognition molecules (PRMs) and enzymes of the lectin pathway and regulatory proteins of the complement system are proposed, and a standardization of language to designate different activation states of complement components is recommended.

## Introduction

**Figure 1:**
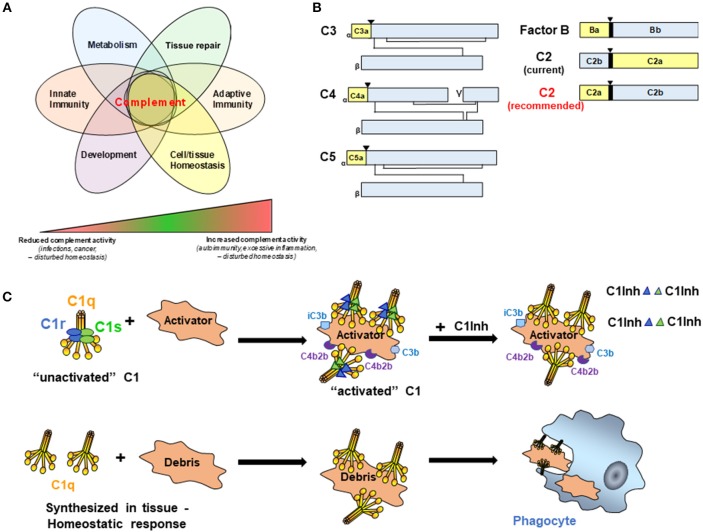
**(A)** Complement activities control key cellular processes and contribute to a broad range of disease states. It is now broadly acknowledged that complement functions well beyond mere protection against invading pathogens but participates actively in the control of key physiological processes. Therefore, complement is key to normal cell and tissue homeostasis and aberrant complement activation (either too little or too much) can hence cause or contribute to a broad range of disease settings including recurrent infections and cancer (too little) or auto-immunity and fibrosis (too much). **(B)** Schematic representation of C3 family members, FB, and C2. While convention holds that smaller fragments (yellow for C3, C4, C5, and FB) retain earlier letters than larger fragments (Blue for C3, C4, C5, and FB), C2 breaks with convention (C2 current). To facilitate communication in the field and better standardize complement nomenclature, we recommend adopting standard convention for C2 (C2 recommended). (▾) Indicates cleavage site for liberation of smaller fragment. **(C)** C1 is a complex macromolecular structure consisting of C1q, C1r, and C1s. C1 circulates in blood as an “unactivated” complex of the recognition protein, C1q (yellow), and two molecules each of the proenzymes C1r and Cls (blue and green ellipses). Conformational changes induced by binding to an activator result in activated C1 due to the conversion of C1r and C1s to active serine proteases (blue and green triangles). C1s proceeds to cleave C4 and C2 which results ultimately in the formation of the classical pathway C3 convertase, C4b2b. Generation of opsonic C3b and iC3b, and subsequently C5a and C5b-9 (not shown) mediate the possible complement effector functions that follow. Four C1-INH molecules per C1 (only representative complexes are shown) are required to inactivate the serine proteases, C1r and C1s, and results in their dissociation from C1q, thereby regulating the amount of C4b2b generated. In the lower row, C1q can be synthesized in tissue to “silently” eliminate apoptotic cells and cellular debris.

The complement system is composed of more than 50 different molecules and cleavage products including but not limited to pattern recognition molecules (PRMs), proenzymes, proteases, anaphylatoxins, opsonins, receptors, regulators, and multi-molecular complexes that are critical to host defense and maintenance of normal tissue homeostasis (1). While traditional functions of the complement system in host defense and clearance of cellular debris have long been appreciated, continued advancement in the field has revealed additional roles for complement from embryogenesis to aging, in both healthy and disease states (Figure 1A) (2–4). In addition, successful development of the anti-human C5 monoclonal antibody, eculizumab, for treatment in paroxysmal nocturnal hemoglobinuria, atypical hemolytic uremic syndrome and refractory myasthenia gravis has renewed interest in clinical applications for complement within the medical field for treatment as well as diagnosis. Consequently, researchers in multiple fields even beyond immunology are investigating various components and pathways of the complement system.

To facilitate advancement and communication in both basic research and clinical application in the field, it is important to standardize complement nomenclature. Following a joint effort of the International Complement Society (ICS) and the European Complement Network (ECN) to simplify and clarify complement nomenclature, a list of recommended names for complement pathways, proteins, protein complexes, and receptors was established in 2014 (5), and the recommendations from that effort are shown in Table 1. This update to complement nomenclature was the first since 1981. While comprehensive, consensus was not reached on several issues. Here we propose recommendations and updates for nomenclature regarding four of these unresolved issues: (1) the cleavage products of C2, (2) C1 complexes- activated molecules, native molecules, or proenzymes, (3) lectin pathway recognition proteins and enzymes and (4) Clusterin.

**Table 1:** (Top) Complement nomenclature as per International Complement Society (ICS) Complement Nomenclature Committee, and ICS and European Complement Network (ECN) boards recommendation from 2014 [reproduced with permission (5)].

**Recommended 2014**		**Recommended 2014**	
**Name**	**Comments**	**Name**	**Comments**
**Pathways**		**Proteins (cont.)**	
CP	Classical pathway	MBL	Mannose-binding lectin
AP	Alternative pathway	Ficolin-1	Ficolin M
LP	Lectin pathway	Ficolin-2	Ficolin L
TP	Terminal pathway (C5, C6, C7, C8, and C9)	Ficolin-3	Ficolin H
**Proteins**		MASP-1	MBL-associated serine protease 1
C1	Complex of C1q, 2C1r, 2C1s	MASP-2	MBL-associated serine protease 2
C1q		MASP-3	MBL-associated serine protease 3
_C1r_		FHL-1	Factor H-like protein 1
C1s		FHR-1	Factor H-related protein 1
C1-INH	C1 Esterase inhibitor	FHR-2	Factor H-related protein 2
C2		FHR-3	Factor H-related protein 3
C3		FHR-4	Factor H-related protein 4
C3(H2O)	Thioester-hydrolyzed form of C3	FHR-5	Factor H-related protein 5
C3a	Anaphylatoxin from C3	CD59	Protectin, Homologous restriction factor
C3b		**Protein complexes**	
iC3b	Inactivated C3b	C5b6	Terminal pathway complex of C5b + C6
C3dg		C5b-7	Terminal pathway complex of C5b6 + C7
C3d		C5b-8	Terminal pathway complex of C5b-7 + C8
C4		C5b-9	Terminal pathway complete complex
C4a		sC5b-9	Soluble C5b-9 with Vn bound
C4a-desArg	C4a without C-terminal arginine	C3bBb	AP C3 convertase
C4b		C3bBbP	AP C3 convertase with properdin
C4d		C3bBbC3b	AP C3/C5 convertase
C4BP	C4b binding protein	C4BP-Protein S	C4BP bound to protein S
C5		**Receptors**	
C5a	Anaphylatoxin from C5	CR1	CD35, C3b/C4b receptor
C5a-desArg	C5a without C-terminal arginine	CR2	CD21, C3d receptor
C5b		CR3	CD11b/CD18 complex
C6		CR4	CD11c/CD18 complex
C7		C3aR	Requesting CD number
C8		C5aR1	C5aR, CD88
C9		C5aR2	C5L2, requesting CD number
Vn	Vitronectin, S protein, S40	CRIg	Complement receptor of the Ig family
FB	Factor B	C1qR	
FD	Factor D	gC1qR	Recognizes globular domains
FH	Factor H	cC1qR	Recognizes collagen domain, calreticulin
FI	Factor I	LHR	Long homologous repeat (CR1)
**UPDATED COMPLEMENT NOMENCLATURE 2019**			
**Proteins**		**Lectin pathway**	
C2a	Small C2 cleavage fragment	CL-10	Collectin-10
C2b	Large C2 cleavage fragment – enzyme	CL-11	Collectin-11
CLU	Clusterin (ApoJ, Sp40,40)	MAP-1	Previously Map44
Properdin		MAP-2	Previously Map19, sMAP
**Protein complexes**			
C1	C1qr_2_s_2_		
Activated C1	Activated complex (containing activated/cleaved C1s)		

*One modification was made to remove 2014 proposed nomenclature for clusterin. (Bottom) (gray): updated complement nomenclature*.

## The Cleavage Products of C2

In general, complement cleavage fragments are designated with letters according to their relative size with “a” fragments smaller than “b” fragments. Figure 1B is a schematic depicting members of the C3 family, as well as Factor B (FB) of the alternative pathway, for which the nomenclature follows this convention. In all of these cases (C3, C4, C5, FB), the larger fragment remains cell associated, and the smaller fragment diffuses from the original site of cleavage. Moreover, C3 family members (C3, C4, and C5) share similar structures, and the smaller cleavage products (C3a, C4a, and C5a) of these molecules all engage receptors on cells to trigger signaling pathways and activation processes. Convention is followed for FB, where the smaller, diffusible fragment, Ba, is liberated from the larger fragment, Bb, which remains associated with activator bound C3b. The serine protease domain of FB is within the Bb fragment.

Current nomenclature in popular use for C2 breaks convention in that the smaller fragment is often referred to as “C2b” and the larger fragment “C2a” (Figure 1B, current nomenclature). Originally, and prior to detailed knowledge of the activation mechanisms involved, the activated C2 molecule was designated as C'2a (6) and refers to the generated ability of C2 to enable/activate the cascade to continue through C3 and ultimately generate a hemolytic activity. However, a challenge to the fragment designation was debated as early as the late 70's, when the protein structure and function clearly showed the lack of conformity with the nomenclature of the other complement activation fragments. Factor B and C2 are homologous proteins [39% sequence similarity (7)], and as such Ba is similar in structure and function to the smaller C2 fragment, and Bb is similar in structure and function to the larger C2 cleavage product. Previous attempts to amend this lack of consistency in the fragment designation [for example, as adopted in Fundamentals of Immunology (8) and editions 1–6 of Janeway's Immunobiology and other texts] were not sustained. We propose that it is time to align the C2 nomenclature with the other complement proteins (Figure 1B, recommended nomenclature). This becomes exceptionally apparent to the student (or instructor) of complement when given the challenge of understanding and communicating a robust system of pathways, receptors and regulators. C2 is clearly the outlier when working through the pathways of complement activation, and it adds ambiguity to a system of pathways that is already challenging to effectively communicate. The argument against adopting the conventional nomenclature for C2 is that it is established in the literature now in a non-conventional format. The counter argument is that it is estimated that our scientific output is doubling approximately every 9 years (9). With the recent resurgence of interest and therapeutic development in the complement field, it will benefit the next generation of complement biologists to learn and work within a system that is “as simple, as clear and as unambiguous as possible” (5).

## C1 Complexes (Unactivated/Activated/Inactivated)

There no longer is a specific designation for the classical complement pathway (CP) proenzymes C1r and C1s or the native zymogen C1 complex vs. activated C1r, C1s, or C1. However, to avoid confusion and therefore facilitate progress toward identification of effective therapeutic targets and therapeutic development, it is critical to accurately describe these various states of the complement components. C1 is a Ca^++^ dependent macromolecular complex comprised of C1q (itself a hexamer of trimers of 3 distinct protein chains C1qA, C1qB, and C1qC), and two molecules each of the proenzymes C1r and C1s (Figure 1C). In blood (or serum), most of the C1q (90%) is found in complex with the proenzymes C1r_2_C1s_2_ (10), and this is “unactivated C1” (or native zymogen C1). That is, under physiologic conditions (vs. a contrived *in vitro* situation), C1q is already complexed to C1r_2_C1s_2_ when it binds to an activator. C1q does not normally bind an activator and then “recruit C1r and C1s,” as has been misstated in recent literature.

When C1q within this C1 binds to “activators,” the C1q molecule is constrained in a conformation that enables C1r and C1s to be cleaved to active enzymes (C1r is autocatalytically cleaved and cleaves C1s). The activated C1s (which now converts the C1 complex to “activated C1”) propagates CP activity by cleaving the next proteins in the cascade as illustrated (Figure 1C). C1 Inhibitor (C1-INH) is an important regulator of this enzymatic activity that rapidly binds covalently in the active catalytic site of each C1r and C1s in the activated C1 complex (i.e., four C1-INH molecules are needed to inhibit the activity of the two activated C1r and two activated C1s molecules per C1 complex). This interaction also mediates dissociation of C1r and C1s from the C1q molecule. However, there is no “inactive” form of C1q. Either after the dissociation of the activated enzymes C1r and C1s from C1q or if synthesized in tissues in the absence of C1r and C1s, C1q has many activities as described in a recent review (11) one of which, the silent clearance of apoptotic cells and cellular debris, is illustrated in Figure 1C.

## Lectin Pathway

The lectin pathway (LP) is activated by multiple PRMs and associated enzymes (12). The PRMs show specificity toward a variety of molecular patterns present on pathogens, but also on endogenous ligands. It is believed that it is the exposure, orientation and spatial distribution of the molecular structure that determines whether binding of the PRMs may lead to complement activation. The PRMs of the LP recognized so far comprise two protein families: the ficolins including ficolin-1, ficolin-2, and ficolin-3, formerly known as M-ficolin, L-ficolin, and H-ficolin, which are encoded by the *FCN1, FCN2*, and *FCN3* genes, respectively, and were previously assigned recommended names as shown in Table 1. The second PRM protein family of the LP is the so-called collectins comprising: mannose-binding lectin (also named MBL or mannan-binding lectin or protein), collectin-10 (also named CL-10, collectin liver-1, or CL-L1) and collectin-11 (also named CL-11 or collectin kidney-1, or CL-K1), which are encoded by the *MBL2, COLEC10*, and *COLEC11* genes, respectively. A large proportion of CL-10 and CL-11 are found as heteromeric complexes in the circulation (CL-10/CL-11 also named CL-LK). We propose that MBL remains as earlier designated and that collectin-10 and collectin-11 designate the latter two members of this family, using the abbreviations CL-10 and CL-11, respectively (Table 1). The above proposals relate to the PRMs of the LP in higher primates, while in lower primates and in other animal species the number and expression of LP PRMs might differ, and the nomenclature may not be directly comparable particularly for the ficolins and for MBL.

The LP PRMs circulate in the blood in complex with associated serine proteases abbreviated MASP-1,−2, and−3 after their original discovery of being associated with MBL. The *MASP1* gene encodes the serine proteases MASP-1 and MASP-3 as well as the non-enzymatically active MAP-1 (also named Map44), while the *MASP2* gene encodes MASP-2 and the non-enzymatically active MAP-2 (also named Map19 or sMAP). MASP-1,−2, and−3 are composed of an *N*-terminal heavy chain and a *C*-terminal light chain containing the serine protease domain, whereas the non-enzymatically active MAPs express unique exons, but only express part of the heavy chains and possess no serine protease domains. The different MASPs and MAPs arise from alternative splicing of the *MASP1* and *MASP2* genes. When the PRMs-MASPs complexes recognize ligands, LP complement activation is subsequently initiated upon MASP-2-mediated cleavage of C4 and C2. MASP-2 was thought mainly to be activated by autoactivation. However, recently it has been shown that MASP-1 may activate MASP-2 and cleave C2, but not C4 and is thus critical in the initiation of the LP. The function of MASP-3 has long been an enigma, but at least one of its functions appears to be cleaving pro-FD to mature active FD enabling activation of the alternative pathway. The MAPs are thought to be regulators of the activity of the LP, but this has so far only been convincingly demonstrated for MAP-1 (Map44). We propose to align the names of the proteins in the lectin pathway of complement with the common nomenclature in the gene databases. The 2014 proposed use of the terms MASP-1, MASP-2, and MASP-3 are reasonable, as are MAP-1 and MAP-2 used for the non-enzymatically active alternative splice variants of the *MASP1* and *MASP2* genes, respectively. However, we also suggest that the abbreviations MASP-1, MASP-2, MASP-3, MAP-1, and MAP-2 in the future will be the names of the proteins without the need for using the “MBL-associated” as that only explains a fraction of their associations.

## Clusterin

Clusterin is a multifunctional glycoprotein known in the complement field as binding to C5b-9 complexes sequestering soluble C5b-9 to prevent host membrane interaction and thus cell and tissue damage (13). However, it is also involved in clearance of misfolded proteins including amyloid ß (14), and clusterin has a risk variant associated with Alzheimer's disease in humans. There are now many publications that abbreviate clusterin as CLU (in line with its gene name, *CLU*), rather than the Cn that was recommended in the 2014 report (5), and CLU was used in the Complement Factsbook 2nd Edition (7) to designate clusterin. Thus, we recommend that the CLU, not Cn, be used as the abbreviation for clusterin.

## Properdin

Properdin, discovered 1954 by Pillemer et al. (15), is currently the only known positive regulator of complement activation. Properdin recognizes and binds to the C3 convertase leading to a 5–10 fold increase in the stability of this enzyme complex (16). Aside from increasing the half-life of the C3 convertase, properdin—after interacting with specific glycosaminoglycans—can also directly initiate complement activation on some altered self-surfaces, such as apoptotic cells, by directing C3b deposition (17). Abbreviating properdin as FP (Factor Properdin) to bring it in line with FB, FD, FH, and FI, was briefly considered among the complement community but was abandoned as the overwhelming majority of publications used, and uses, the term properdin without any arising issues. Thus, we recommend that properdin be used as the sole term.

To conclude, we hope that the updates proposed here for cleavage fragments, PRMs, activation states and regulatory proteins of the complement system will enhance communication and thus understanding of both the basic complement pathways and consequences of activation or lack thereof, as well as, the newly discovered nuances of the complement system in the classroom, in research, in pharma and in the boardroom. It is hoped that the simplified uniform nomenclature of this intricate system will facilitate therapeutic development and appropriate application to the clinic. We propose that the ICS consider and endorse these changes and submit them to the IUIS Nomenclature Committee.

## Author Contributions

AT initiated work. SB, PG, CK, and AT contributed to draft, revision, and approval of final version.

### Conflict of Interest Statement

The authors declare that the research was conducted in the absence of any commercial or financial relationships that could be construed as a potential conflict of interest.
